# Temporal evolution of the environmental footprints of intensive farming across England

**DOI:** 10.1371/journal.pone.0346664

**Published:** 2026-04-29

**Authors:** Yusheng Zhang, Adrian L. Collins

**Affiliations:** Rothamsted Research, North Wyke, Okehampton, Devon, United Kingdom; University of Kalyani, INDIA

## Abstract

The agriculture sector continues to face expectations regarding documenting its unintended environmental footprints through time given increasing awareness of the need to deliver food more sustainably. Accordingly, the work reported in this paper used a process-based modelling framework evaluated previously to examine the temporal evolution (2010, 2016, 2021) of the environmental footprints of intensive farming systems across England. The results were computed at Water Management Catchment scale given the use of this spatial unit for policy reporting purposes. Environmental footprints focused on Global Warming Potentials over 20- (GWP20) and 100-year (GWP100) horizons, eutrophication potential (EP) and acidification potential (AP). Both GWP20 and GWP100 were included to characterise the short- and long- term impacts. Important structural changes in agriculture included a 3.7% increase in the area of land under ‘general cropping’ and a 2% reduction in that under dairy. There was a corresponding reduction of the registered cattle populations by 4% in 2016 and 12% in 2021, relative to 2010. At the same time, the number of sheep and lambs increased by 9% in 2016 and 4% in 2021. These farm structure changes in 2021 were computed to result in respective median reductions in GWP100, EP and AP of 18%, 13% and 21% compared with 2010. Eutrophication potential exhibited lower overall reductions wherein 28 water management catchments exhibited <5% reductions, with a maximum reduction of ~45%. In comparison, corresponding maximum reductions in GWP20, GWP100 and AP were modelled at 76%, 64% and 54%, respectively, with 17, 15 and 7 water management catchments exhibiting <5% reductions. Modelling suggests a downward change in environmental footprints but there are challenges in directly linking predicted temporal trends with existing and available monitored data.

## 1. Introduction

To meet global challenges surrounding food security, climate change and biodiversity decline, the agriculture sector needs to mitigate the unintended environmental impacts resulting from the exploitation of natural resources, perturbations to interlinked biophysical processes, and emissions of harmful substances into various components of the earth’s system, including air, water and soil [1]. Importantly, the existing damaging environmental footprints of farming will impede the achievement of various United Nation’s Sustainable Development Goals [2] and national policy outcomes focussed on those goals.

Because of the reliance of agriculture on the natural environment, as well as industrial processes and human activities, the environmental footprints associated with intensive farming do vary with time and different farm systems. Intensive farming demands high inputs of fertilisers and pesticides, and use of advanced technologies. Here, changing climate conditions [3], volatile energy and agricultural product markets [4], evolving land use, better field and farm management practices and improving production efficiency within supply chains all play a role in shaping the environmental footprints of agriculture [5]. At the same time, the increased awareness of environmental issues associated with farming at local and global scales, has driven various regulatory directives (e.g., The Nitrates Directive in the UK (91/676/EEC)), agri-environment schemes (e.g., [6]) and voluntary actions for farmer win-wins and all of these can influence farmer behaviours in the sense of being more environmentally friendly.

The environmental footprints of agriculture can be characterised in different ways. The simplest approach involves the reporting of key pollutant loadings from agricultural land [7,8] wherein the selection of priority pollutant types could be site-specific and policy objective oriented. These pollutant loadings can be compared against known environmental pressures at local and global scale to generate environmental impact indices [9,10]. Increasingly, such indices are combined with standardised characterisation factors (CF) to estimate mid-point Life Cycle Assessment (LCA) environmental impacts, such as Global Warming Potential (GWP), eutrophication potential (EP) and acidification potential (AP) [11]. Such impacts can be extended further using embedded nutrition values for supporting broader comparisons among different products or farm systems [12]. Recent work has also demonstrated how integrating process-based modelling and LCA can support the spatial mapping of mitigation potentials for the environmental footprints of agriculture and different farm systems therein [13].

Turning specifically to the UK, earlier work on the environmental footprints of agriculture has focused on the carbon footprints of specific farm businesses, such as broiler [14], sheep [15], or lamb and beef [16] systems. More recently, pollutant emissions for multiple representative farm types across England and Wales have been reported and their relationships with farm production examined [17]. For emissions to air, the annual emissions of greenhouse gases (GHG) from the agricultural sector, including methane (CH_4_) and carbon dioxide (CO_2_), as well as other priority gases such as ammonia (NH_3_), has been updated and mapped as part of the long-running national inventory submissions [18]. However, one important limitation here is that the reported sector and national totals by various categories have not been spatially disaggregated and this research gap requires attention. To date, mapped gaseous emissions only have limited categories because of potential data disclosure issues. As a result, and to deliver novelty in this research space, more comprehensive and up-to-date information for management scales including farm, catchment and regional, are needed for the development of cost-effective and targeted, equitable mitigation strategies. Furthermore, explicit assessment of temporal changes in the environmental footprints could provide further insights into the impacts of various controlling and/or regulating factors in either natural or human systems, thereby delivering additional novelty.

Accordingly, the work herein adopted a modelling approach and used spatially explicit data on the natural environment and farming activities to quantify the environment footprints of intensive farm types in three deep dive years (2010, 2016 and 2021). Between 2010 and 2021, the agricultural sector in the UK has experienced a food crisis [19], fluctuating fertiliser and grain prices [4] and migration of environmental schemes embedded within national agricultural policy [20]. These significant changes were expected to have significant impacts on environmental footprints. Using our established modelling framework, our research aimed to quantify the technically feasible changes in multiple indicators concerning environmental implications at management scales.

## 2. Methodology

The quantification of environmental footprints was achieved with the implementation of a farm to landscape multipollutant modelling framework; namely, the Catchment Systems Model (CSM) [21]. Predictions generated by routines embedded in the framework have already been validated with monitored data at field [22] and national scale [8] and used for the impact analysis of mitigation measures [8], scaling out experimental outputs [22] and for scenario modelling at catchment scale [11]. More details about the model, including modules, data flows and a list of the on-farm mitigation measures included is available [21].

**Fig 1:**
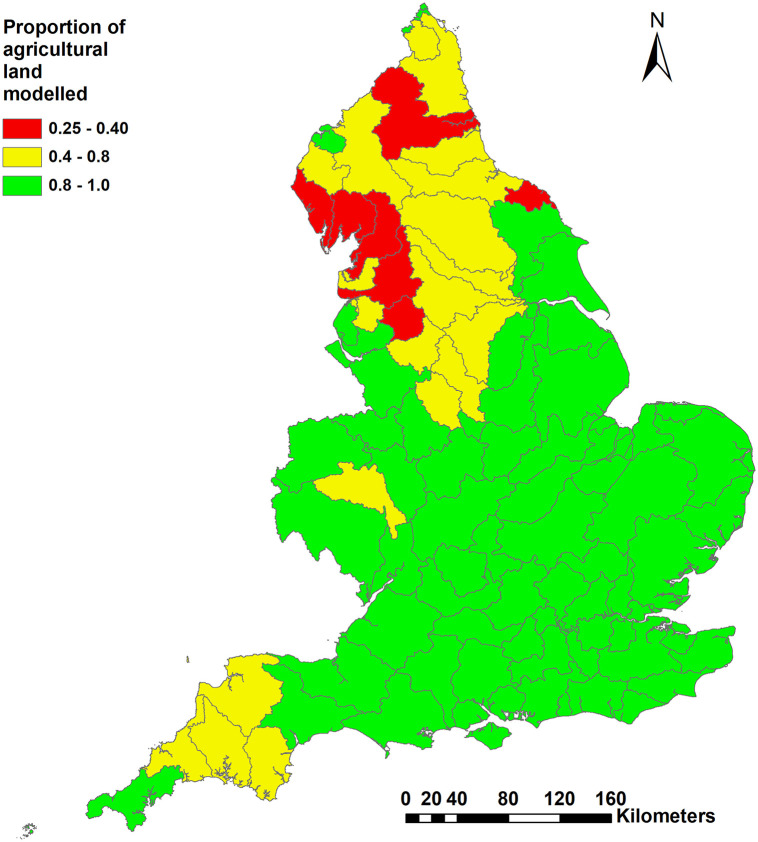
Water Management Catchments across England and the mapped coverage of farmland modelled in each (contains public sector information under the Open Government License 3.0).

The selected spatial modelling unit was focussed on so-called Water Management Catchments (WMCs; Fig 1). The WMC is one of the management units related to Water Framework Directive (WFD) reporting whilst the UK was a member of the European Union, but which has been continued as a policy reporting unit since the exit decision. Representative model farms were generated within each WMC to reflect the robust farm types [23] present involving the most intensive land management with high economic inputs, comprising cereal, dairy, general cropping, horticulture, lowland grazing livestock (lowland grazing hereafter) and mixed farms. Less favoured area (LFA) grazing livestock, specialist pig and specialist poultry farms were excluded. These exclusions were considered to have limited impact on the estimates at national scale due to either their extensive management practices (e.g., LFA grazing) where low agricultural inputs are expected or relatively small numbers of holdings and limited land use resulting in their contributions to overall agricultural environmental footprints being insignificant. This might not be the case for some specific WMCs but was adopted to rationalise the national scale modelling exercise. The proportion of agricultural land modelled on the basis of the selected intensive farm types in each WMC is illustrated in Fig 1.

Each model farm in CSM has a unique combination of total annual rainfall, soil properties based on the Hydrology of Soil Types classification scheme [24] for derived soil drainage status and farm type specific cropping areas and livestock populations if present. Considering the availability and accessibility of the agricultural census data for England, the years 2010, 2016 and 2021 were selected for this study. Aggregated June Agriculture Survey data for these years at waterbody scale were sourced from the UK Department for Environment, Food and Rural Affair (Defra). The holding numbers by farm types and the locations of individual farms were available. The latter were used to calculate the frequency distribution of farms in different combinations of rainfall and soil zones. The rainfall data were based on the 3-year average of mapped annual rainfall [25] around the selected modelling years; i.e., the preceding year, modelling year and following year. It was assumed that there were no changes in soil structure or associated drainage status between the modelling years.

To represent changes in management practices, data from annual national surveys, such as the British Survey of Fertiliser Practice (BSFP; [26]) and Farm practice survey [27] were consulted. Similar to the procedures used in the estimation of annual rainfall, crop-specific fertiliser application rates around the three individual modelling years were used to specify nitrogen and phosphorus input rates for the model farms. Data for ‘Other livestock’ was used as the input values for lowland grazing farms. No regional variations in application rates for specific crops on specific farm types were considered. No changes of soil drainage status over the modelling period (2010 to 2021) were represented so the effects of localised tile drain installations could be overlooked. No evidence indicates that this would affect the assessment of temporal changes at national scale. To simplify the modelling procedure, it was assumed that there is no manure export or import from the model farms. Furthermore, manure spreading preferences for different crops and grassland were not changed between the modelled years. Examination of BSFP data for the relevant years supported this assumption. To account for potential impacts arising from regulatory designations, official maps of the spatial extent of Nitrate Vulnerable Zones in 2013, 2017 [28] and 2021 [29] were used for 2010, 2016 and 2021, respectively. This decision reflected the fact that NVZ designations are reviewed and updated every four years, resulting in some temporal mismatches between decisions surrounding designations and our selected deep dive modelling years; e.g., for 2010. Regional crop yield time series for wheat, winter barley, spring barley and winter OSR [30] (were downscaled to WMC scale to parameterise CSM. No temporal variations of the yields for other crops, which account for <30% of total cropping areas, were considered. To account for the effects of on-farm mitigation measures for the different modelled years, changing uptake rates of individual measures were based on annual farm practice surveys [27] data on uptake rates included in other tools [31] and previous policy-focused work [8,11]

For the calculation of GHG emissions, embedded emissions associated with the off-farm manufacturing of input products (e.g., fertilisers) were included. The CO_2_-equivalent emissions from the use of various fuels and electricity, were estimated with the recommended conversion factors defined by the UK government [32]. For fertilisers, which are dominated by ammonium nitrate and urea, regional-specific estimates from published literature were used. These included those from Marinussen et al. [33] for 2010, Hoxha and Christensen [34] for 2016 and the Vidovic et al. [35] for 2021, respectively. The actual values used are provided in Table S1 in the supplementary information [36].

LCA-based mid-point impacts were calculated using the following formulas:


$$GWP\text{2}\text{0}=\text{8}\text{1}.\text{2}*{CH}_{\text{4}}+\text{2}\text{7}\text{3}*\;N_{\text{2}}O$$

$$GWP\text{1}\text{0}\text{0}=\text{2}\text{7}.\text{9}*{CH}_{\text{4}}+\text{2}\text{7}\text{3}*\;N_{\text{2}}O$$

$$AP=\text{1}.\text{6}*{NH}_{\text{3}}+\text{0}.\text{5}*\;N_{\text{2}}O$$

$$EP=\text{0}.\text{1}*{NO}_{\text{3}}+\text{3}.\text{0}\text{6}*P+\;\text{0}.\text{3}\text{5}*\;{NH}_{\text{3}}$$

where: CH_4_, N_2_O, NH_3_, NO_3_ and P are methane, nitrous oxide, ammonia, nitrate and phosphorus loads in kg or kg ha^-1^, respectively. The specification of coefficients for GWP20 and GWP100 calculations were based on recommended values in the IPCC Sixth Assessment Report [37]. The characterization factors used for EP and AP were taken from CML-IA 2016 [38].

To explore temporal variations, the coefficients of variation (CV) among the three modelled deep dive years, were calculated by treating each year as an independent sample for each WMC. There is no context-specific criterion to categorise the magnitude of CV, but it is suggested that a widely used classification scheme for the grouping of spatial variability in soil science [39] could be adopted here; i.e., < 15% for low variation, 15–35% for moderate variation and >35% for high variation.

For the evaluation of modelled water quality related pollutant emissions, monitored nitrate, suspended solid and total phosphorus concentrations across England [40] for the 3 simulation years were downloaded, filtered and processed to obtain monthly estimates. The selection process ensured that all selected sites are within the WMCs and have at least 10 valid monthly data points from routine monitoring for each modelled year. The exclusion of monitored data with <10 months of valid observations ensured the robust estimation of valid individual annual means. Visual examination suggest that enforcement of this restriction has no undue influence on any regions across England. It has to be acknowledged that this does reduce the number of valid annual averages available for the generation of summary statistics. The full datasets, including excluded data points, can be found in Supplementary information [36].

## 3. Results

The modelling exercise covered >72000 km^2^ of agricultural land across England. Inter-annual variations between the total area of modelled land for the selected deep dive years were only ~1%.

### 3.1 Significant structural changes in agricultural land use and management

**Fig 2:**
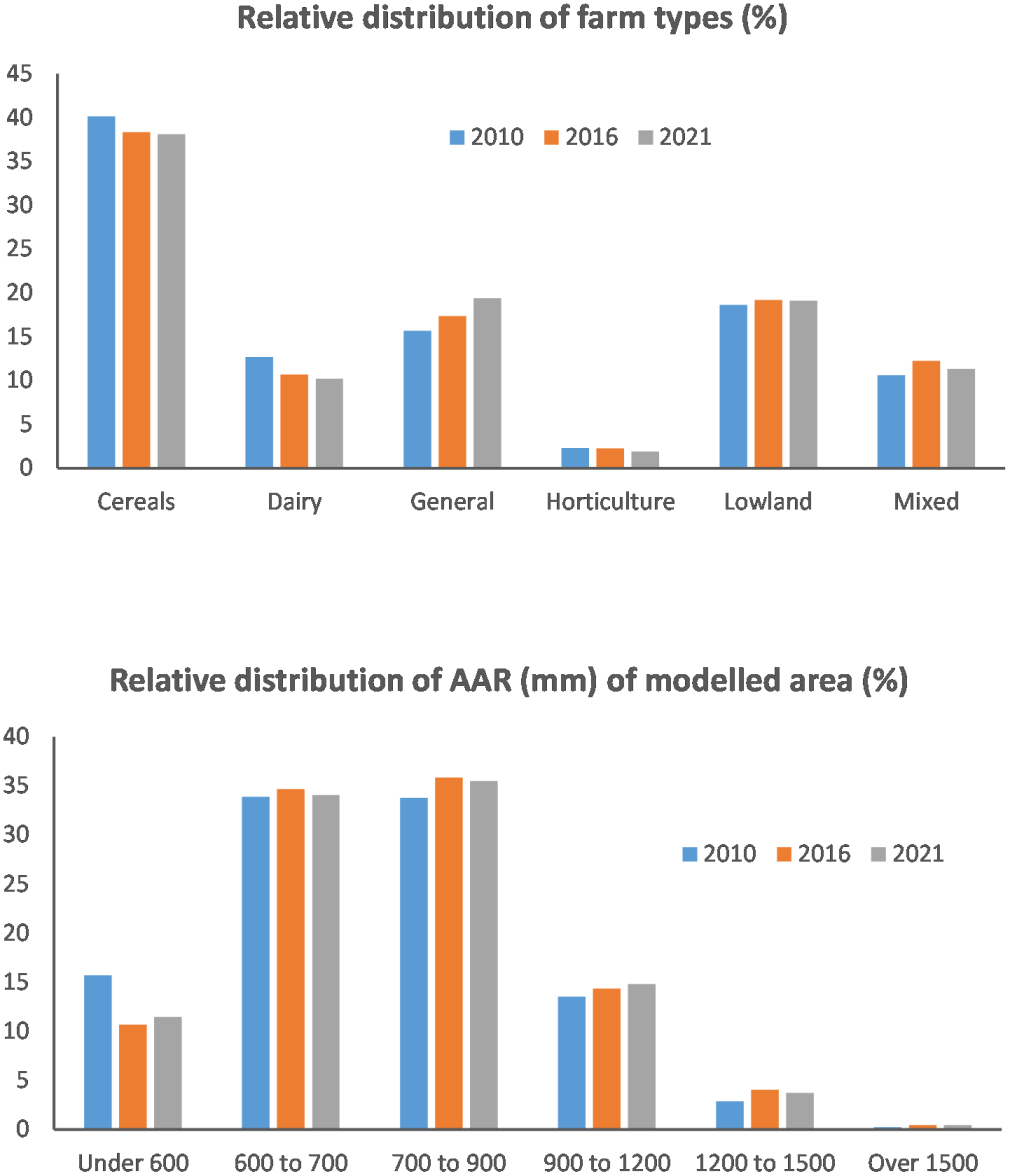
Frequency distributions of farm types and annual average rainfall for the three modelled years.

Frequency analysis of farm type distribution by land areas and their geographical locations in the different rainfall bands recognised by CSM for each of the modelled years are shown in Fig 2. The reported cropping areas and livestock populations for the modelled areas in 2010 and the relative changes in 2016 and 2021 are summarised in Table 1. The results indicate that the most significant change concerned the gradual increase (3.7%) of land under ‘general cropping’ and a reduction (2%) in the area under dairy, especially for the period between 2010 and 2016. The shrinkage of dairy farming was also confirmed by the reduction of the registered cattle populations by 4% in 2016 and 12% in 2021, relative to 2010. At the same time, the number of sheep and lambs increased by <10% in 2016 but fell back to a < 5% increase in 2021. National statistics for the UK dairy industry [41] suggest that the reduction of dairy cow counts was compensated by increases in milk yield per head.

**Table 1:** Structural changes for the modelled farms associated with cropping areas and livestock populations for the modelled years.

Crop	Area in 2010 (ha)	2016 vs 2010 (ratio)	2021 vs 2010 (ratio)
Permanent Pasture	2497300	0.96	0.92
Rotational Grassland	541110	1.08	1.30
Winter Wheat	1754344	0.94	0.92
Winter Barley	308992	1.17	1.07
Spring Barley	256658	1.58	1.78
Winter OSR	633170	0.89	0.48
Maize	142979	1.24	1.43
Potatoes	98540	1.04	1.02
Sugar Beet	115640	0.73	0.80
Peas and beans	198419	1.11	1.21
Crops for stockfeed	25464	1.64	2.19
Minor cereals	23225	1.65	2.55
Vegetables	102588	0.89	0.85
Fruits	29721	1.06	1.02
Uncropped arable land	141045	1.49	1.58
Farm woodland	264314	1.24	1.28
Livestock	Head counts in 2010	2016 vs 2010	2021 vs 2010
Cattle	4769800	0.96	0.88
Sheep and lamb	8248118	1.09	1.04

There are also noticeable changes in annual rainfall among the three modelled years. More farms (15.7%) were in the lowest rainfall band in 2010 and less in the two other years (~11%). Only 3.1%, 4.4% and 4.1% of modelled farmland experienced annual rainfall >1200 mm in 2010, 2016 and 2021, respectively. For England as a whole, the mapped rainfall distributions suggest that 2010 was a relatively drier year whilst 2016 was the wettest among the modelled years. As expected, there were no systematic changes in the distribution of farmed areas in soils with different drainage status wherein ~60% of farmed areas were in either drained or heavily drained soils and 40% in free draining soils. Examination of crop-specific areal changes showed some distinctive temporal patterns. While permanent grassland areas decreased by <1% a year, rotational grassland areas have increased, especially since 2016. It can be seen that the spring barley, maize, peas and beans, crops for stockfeed and minor crops growing areas continued increasing across the modelled years by varying degrees, whereas winter OSR areas continued decreasing. In term of actual land areas affected for England as a whole, there has been a significant decrease for winter oilseed rape, permanent grassland and winter wheat, but considerable increases for spring barley and rotational grassland (Table 1). There was also evidence for increased specialisation and intensification as, relatively speaking, more arable crops were in cereal farms, more cattle on dairy farms and more sheep and lambs on lowland grazing farms by 2021.

Nitrogen application rates for different crops and land use are one of the most important and dynamic inputs for farming as they are significantly correlated with both crop yields and emissions intensities. The annual rates for major farm types used for 2010 and the ratios of the corresponding rates for the other two modelled years, relative to 2010, are tabulated in Table 2. Overall, there was an increase in 2016 followed by a decrease in 2021 for most crops/ land uses. The application rates for potatoes decreased whilst the rates for soft fruits increased. Between 2016 and 2021, there was also a notable decrease in application rates for permanent grass, sugar beet, orchards and brassica vegetables. The reasons for this change are not clear. The tightening of environmental regulations, introduction of various agri-environment schemes, improved land management practices (e.g., precision farming), and the adoption of new crop varieties with higher nitrogen use efficiency could all contribute. The trends since 2010 were clearly crop/ land use, farm type and year dependent. Examination of fertiliser application timings suggest that no significant changes were reported as ~80% of fertilisers were applied between March and May with 26–29% in March, 36–39% in April and 15–17% in May for all three modelled years. Similarly, manure application rates and coverage did not exhibit significant changes among the modelled years.

**Table 2:** Annual nitrogen fertiliser application rates (kg ha^-1^) in 2010 and relative ratios for the other two modelled years (2016 and 2021) for selected crops and land uses.

Crop/ land use	Application rates in 2010			2016 vs 2010			2021 vs 2010		
	Cereal	Dairy	Lowland*	Cereal	Dairy	Lowland*	Cereal	Dairy	Lowland*
Permanent Pasture	34	114	35	1.02	0.97	0.96	0.89	0.85	0.80
Rotational Grassland	71	137	80	1.20	1.08	1.00	1.13	0.98	0.84
Winter Wheat	200	159	165	0.96	1.06	0.79	0.92	0.95	0.97
Winter Barley	147	127	105	1.02	1.10	1.25	0.95	0.87	1.24
Spring Barley	109	79	73	1.03	0.94	1.12	0.96	0.79	1.03
Winter OSR	197	190	151	0.93	1.03	1.22	0.83	0.65	1.09
Maize	68	40	33	1.17	1.21	1.12	0.97	1.32	1.05
Potatoes	171	156	156	0.76	0.91	0.91	0.62	0.85	0.85
Sugar Beet	98	92	92	0.95	1.04	1.04	0.69	0.71	0.71
Fodder Crops	54	66	50	1.25	0.94	1.09	1.07	0.66	0.86
Other Crops	69	69	58	0.99	0.91	1.08	1.20	0.99	1.21
Vegetables (Other)	63	57	90	0.91	0.84	0.54	1.00	1.28	0.81

* Lowland stands for lowland grazing.

Spatial mapping indicated that 67.6% of the modelled farming area was located inside the NVZ areas in 2010, reducing slightly to 66.9% and 66.2%, in 2016 and 2021, respectively. The changes were therefore small and very localised. Based on available survey reports and expert knowledge about the status of active agri-environment schemes, the following on-farm measures were judged to have evidenced step changes in their implementation levels across England between 2010 and 2021:

• Establish cover crops in the autumn
• Cultivate compacted tillage soils
• Cultivate and drill across the slope
• Establish in-field grass buffer strips
• Loosen compacted soil layers in grassland fields
• Reduce field stocking rates when soils are wet
• Incorporate manure into the soil

### 3.2 Modelled key pollutant loadings to water and air at national scale

Using temporal and spatial specific parameterisation of CSM, representative farms across the different WMCs were created and farm scale emissions to water and air were calculated for the three modelled years. Combining WMC and farm type specific holding counts and average farm areas, total and specific loadings for each WMC and the whole of England were estimated (Table 3).

**Table 3:** Estimated key agricultural pollutant loadings and emissions, carbon stocks and farm energy use in 2010, 2016 and 2021 at national scale.

Pollutant	2010 load	2016 load	2021 load	National totals in 2010	2016 vs 2010	2021 vs 2010
	(kg ha^-1^)	(kg ha^-1^)	(kg ha^-1^)	(tonnes)	(ratio)	(ratio)
Nitrate	22.8	22.1	20.5	166347	0.97	0.89
Phosphorus	0.45	0.47	0.45	3273	1.06	0.99
Sediment	216.5	245.6	243.0	1579577	1.13	1.11
Ammonia	10.9	9.5	8.9	79460	0.87	0.81
Methane	60.0	52.8	49.9	438089	0.88	0.82
Nitrous Oxide	2.50	2.33	2.12	18275	0.93	0.84
Soil Carbon stocks	144.1	143.6	142.8	1051147	1.00	0.98
Energy Use (CO_2_e)	1215.9	792.1	687.0	8871395	0.65	0.56

The results in Table 3 suggest that most loadings exhibited a continuous decreasing trend. These reductions were manifested in the case of nitrate and phosphorus to water and methane, nitrous oxide and ammonia to air. Carbon stocks remained almost unchanged across the modelled years, but much higher agricultural sediment loads were reported in 2016 and 2021 with an estimated increase of 13% and 11%, respectively, compared to 2010. These increases were mainly on lowland grazing and mixed farms for 2016 and lowland grazing, general cropping and cereal farms in 2021 where they all contribute to >20% of the increases reported at national scale. Some noticeable changes were computed for 2016, wherein the emissions from farm energy use reduced by 35% and methane and ammonia emissions reduced by >10%, compared with 2010 (Table 3). Farm energy use covered a wide range of in-field and on-farm activities, including cultivation, planting, irrigation, spreading of fertiliser and manure, application of plant protection products (PPPs), harvesting, grain drying and storage, as well as on-farm livestock management, such as feeding, housing, cleaning and milking. It also included the management of slurry, farmyard manure (FYM), litter and dirty water as well as embedded emissions associated with the production and transport of fertilisers and PPPs.

### 3.3 Changes in environmental footprints

To represent the interactions among different pollutants and their overall environmental footprints, GWP20, GWP100, EP and AP at national scale for the three modelled years were calculated and the national totals for different intensive farm types were scaled by corresponding farm areas. The results for 2010 along with their relative changes for the other two modelled years are presented in Table 4. The significant contribution of livestock farms, especially dairy farms, is clearly evident. It is estimated that the relative contributions of dairy farms to the overall reduction, between 2016 and 2010, of GWP20, GWP100, EP and AP are 70%, 66%, 84% and 63%, respectively. For the reduction between 2016 and 2021, the corresponding relative contributions are lower, ranging between 47% to 56% for the four indicators considered. For cereal farms, a significant reduction in GWP20, GWP100, and AP were observed in 2021 but not in 2016. Apart from EP, lowland grazing and mixed farms have higher reductions in 2016. Clearly, there are some farm type specific trends illustrated in Table 4.

**Table 4:** Estimated areal specific environmental footprints in 2010 and relative changes in 2016 and 2021.

Farm type	GWP20 in 2010	2016 vs 2010	2021 vs 2010	GWP100 in 2010	2016 vs 2010	2021 vs 2010
	(kg ha^-1^ yr^-1^)	Ratio	Ratio	(kg ha^-1^ yr^-1^)	Ratio	Ratio
Cereals	871	1.09	0.93	712	0.99	0.87
Dairy	22469	1.00	1.01	8541	1.00	1.01
General cropping	469	1.62	1.39	469	1.17	1.01
Horticulture	400	2.81	2.23	400	1.84	1.36
Lowland grazing	8493	0.84	0.80	3284	0.85	0.81
Mixed	6623	0.78	0.75	2810	0.80	0.76
All farms	5559	0.89	0.83	2359	0.89	0.84
	**EP in 2010**	**2016 vs 2010**	**2021 vs 2010**	**AP in 2010**	**2016 vs 2010**	**2021 vs 2010**
	**(kg ha**^**-1**^ **yr**^**-1**^)	**Ratio**	**Ratio**	**(kg ha**^**-1**^ **yr**^**-1**^)	**Ratio**	**Ratio**
Cereals	5.3	0.98	0.90	9.3	0.91	0.80
Dairy	19.9	0.99	1.00	65.8	0.98	1.00
General cropping	4.5	0.95	0.84	6.2	1.01	0.84
Horticulture	4.0	1.67	0.92	5.4	3.01	0.98
Lowland grazing	5.9	0.99	0.93	16.4	0.88	0.83
Mixed	8.6	0.83	0.86	23.1	0.71	0.77
All farms	7.5	0.94	0.88	18.7	0.88	0.82

The estimated national total footprints for different farm types in 2010 and their relative changes in 2016 and 2021 are shown in Table 5. It is estimated that dairy farms contributed to 51%, 46%, 30% and 45% of GWP20, GWP100, EP and AP of all the farms modelled. Cereal farms had more impact on EP (29%), followed by AP (20%), GWP100 (12%) and GWP20 (6%). By contrast, lowland grazing farms contributed more to GWP20 and GWP100 (26–28%) and less to EP and AP (~15%). Mixed farms delivered ~13% of emissions for the four indicators under examination. The relative contributions from general cropping and horticulture farms rarely exceeded 5%, with the most significant impacts on EP. Readers are reminded that these estimates are a function of areal specific emissions, farm areas and the number of holdings for each farm type.

**Table 5:** Estimated national totals of environmental footprints in 2010 and their relative changes in 2016 and 2021.

Farm type	GWP20 in 2010	2016 vs 2010	2021 vs 2010	GWP100 in 2010	2016 vs 2010	2021 vs 2010
	kilotons	Ratio	Ratio	kilotons	Ratio	Ratio
Cereals	2551	1.04	0.88	2086	0.94	0.82
Dairy	20749	0.84	0.81	7887	0.84	0.81
General cropping	537	1.79	1.70	537	1.29	1.24
Horticulture	67	2.73	1.84	67	1.78	1.12
Lowland grazing	11536	0.87	0.81	4461	0.88	0.82
Mixed	5123	0.90	0.79	2173	0.92	0.81
Grand Total	40562	0.88	0.82	17212	0.89	0.83
	**EP in 2010**	**2016 vs 2010**	**2021 vs 2010**	**AP in 2010**	**2016 vs 2010**	**2021 vs 2010**
	**kilotons**	**Ratio**	**Ratio**	**kilotons**	**Ratio**	**Ratio**
Cereals	15.61	0.93	0.85	27.4	0.87	0.75
Dairy	18.38	0.83	0.79	60.7	0.82	0.80
General cropping	5.18	1.05	1.03	7.1	1.12	1.02
Horticulture	0.67	1.62	0.75	0.9	2.92	0.80
Lowland grazing	7.96	1.02	0.94	22.3	0.90	0.84
Mixed	6.65	0.96	0.91	17.9	0.81	0.81
Grand Total	54.46	0.93	0.87	136.3	0.87	0.81

The analysis suggested the median CV for the modelled farm areas among all WMCs was 4.7%, with an inter-quartile range (IQR) of 6%. For the estimated totals for GWP20, GWP100 and AP, the corresponding median CVs ranged between 11% and 12% with the IQR varying between 6% to 9%. EP exhibited a slightly lower median CV and IQR at 8% and 6%, respectively.

**Fig 3:**
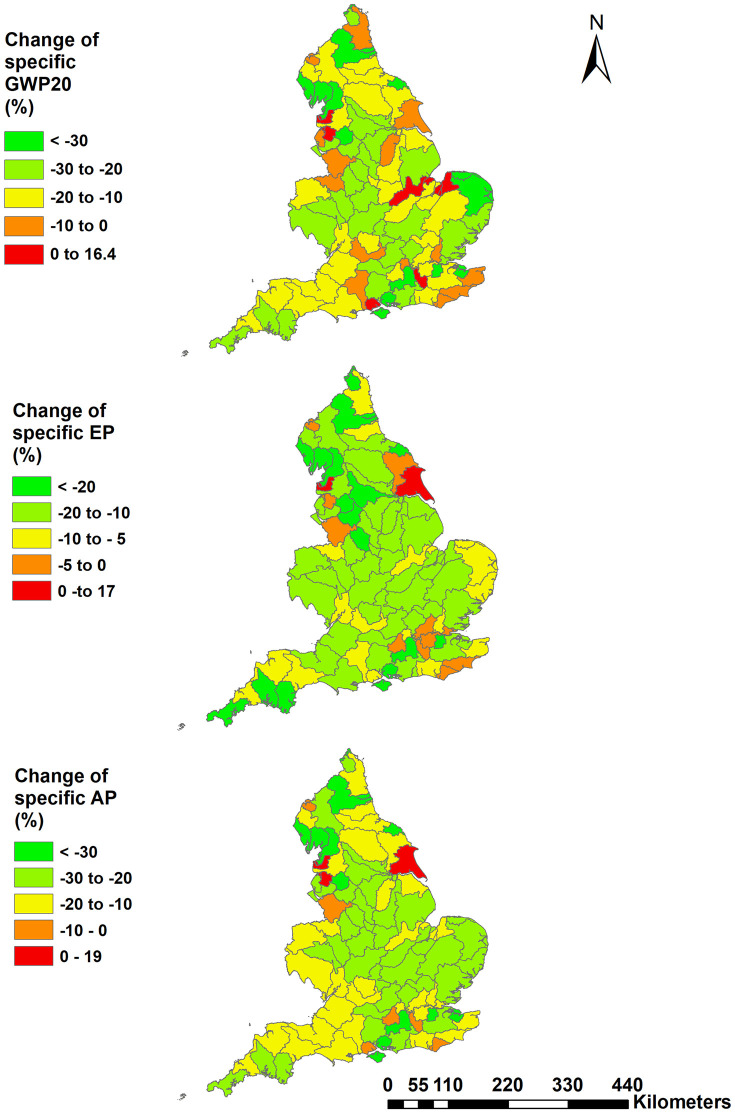
The spatial patterns of relative changes in areal specific GWP20, EP and AP in 2021, relative to 2010 (contains public sector information under the Open Government License 3.0).

To further examine the spatial patterns of change at WMC scale, the relative changes of GWP20, EP and AP in 2021 relative to 2010 are mapped in Fig 3. There are a few WMCs which have increased environmental footprints from agriculture: 6 for GWP20, 4 for GWP100, 2 for EP and 3 for AP. Overall, however, most of the WMCs exhibited lower agricultural environmental footprints in 2021, compared with 2010. Here, the estimated respective median reductions were 18%, 18%, 13% and 21%, respectively. Compared with the other footprint indicators, EP manifested a more distinctive distribution as it exhibited lower overall reductions; 28 WMCs have <5% reduction and the maximum reduction was computed to be ~ 45%. In comparison, 17, 15 and 7 WMCs exhibited <5% reductions with corresponding maximum reductions modelled at 76%, 64% and 54% for GWP20, GWP100 and AP, respectively (Fig 3).

## 4. Discussion

The environmental footprint of intensive farming affects multiple components of agroecosystems, including the pedosphere at field scale, waterbodies at catchment scale and the atmosphere at global scale. The consideration of multiple mid-point impact indicators, comprising GWP, EP and AP, facilitates a comprehensive assessment of potential unintended consequences. Our modelled outputs demonstrate both temporal and spatial variations. These patterns can be attributed to interacting factors, including changing weather conditions represented by annual average rainfall, land use patterns represented by different cropping areas and livestock populations, adaptive management practices represented by fertiliser application rates and the implementation of mitigation measures driven by regulation, incentivization and on-farm advice. CSM provides a basis for examining potential trade-offs and co-benefits at appropriate management scales, starting with farms and then scaling up to WMCs and national level.

The reported significant structural changes in agricultural land use and management can be attributed to the complex interactions of changing climate, market volatility from geopolitical events [42], and farmer participation in various environmental schemes. The temporal trend observed in fertiliser use is primarily due to the rising costs of energy and high demand during Covid recovery in 2021 [43], which resulted in higher fertiliser prices and lower application rates. The most significant change identified by the modelling exercise herein is the continued reduction of the environmental footprints associated with energy use but the rate of reduction has slowed down as shown in Table 3. Here, the national loads reduced by >30% between 2010 and 2016 but only by <10% between 2016 and 2021. It is suggested that the most significant change reported for energy use in 2021 relative to 2010 mainly resulted from the reduced energy density of electricity, ammonium nitrate and urea, which have decreased by around 60%, 58% and 17%, respectively. For the same period, a reduction of energy intensity for the agriculture, forestry and fishing sector as a whole was also reported by the Office for National Statistics, UK [4]. The reported trend here is also in line with assessments by the fertiliser industry which argues that significant progress has been made in the lowering of emissions from fertiliser production processes but that we are approaching the technical limits with existing technologies and infrastructure [5].

**Fig 4:**
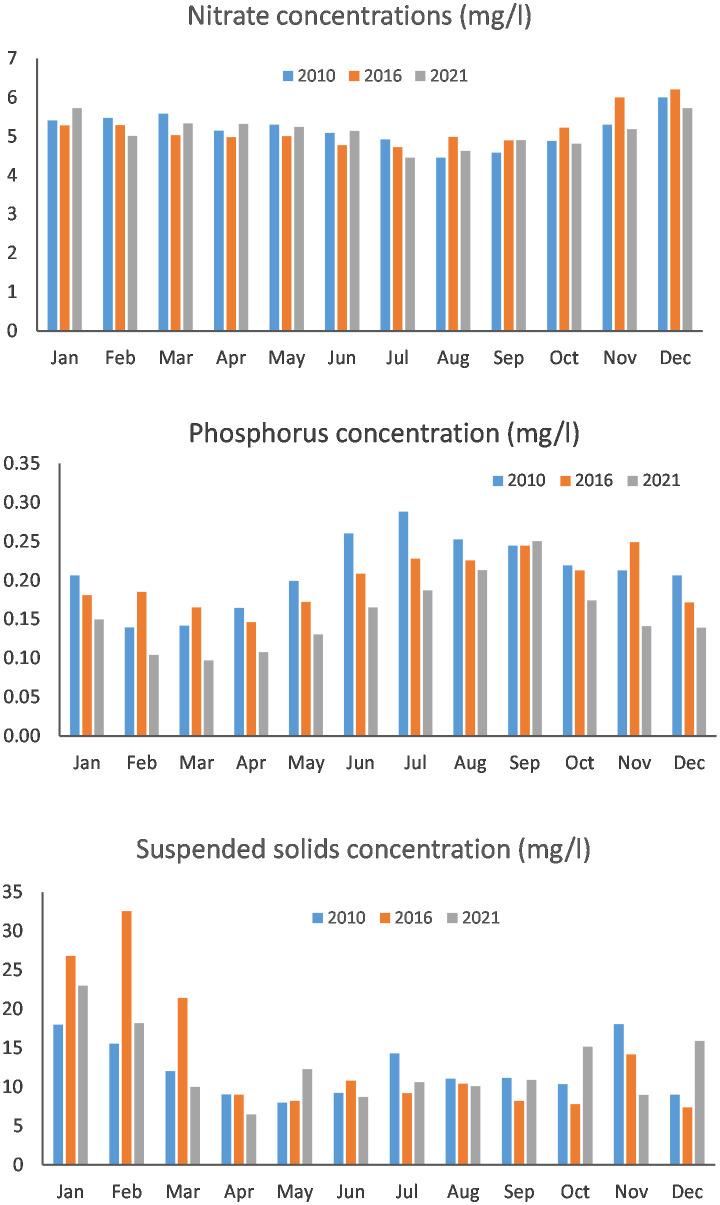
Monthly average concentrations of nitrate, phosphorus and suspended solids in the three modelled years based on filtered monitoring data from the UK water quality data archive (https://environment.data.gov.uk/water-quality/view/landing).

GHG inventory reporting for England [18] has revealed similar downward trends in 2021 for agricultural related emissions to air (i.e., ammonia, methane and nitrous oxide) but the reported magnitudes are smaller with reductions being ~5% to 7% instead of the 16% (i.e., ratio of 0.84 relative to 2010) to 18% (i.e., ratio of 0.82 relative to 2010) predicted by our work and shown in Table 3. The discrepancy here can be partially explained by the different assumptions about the mitigation measures implemented. Only a limited number of measures were considered for the GHG inventory reporting [18], whereas our modelling included a more comprehensive list. For emissions to water, the monitored concentrations of nitrate, phosphorus and sediment in rivers across England [40] in the targeted modelling years are shown in Fig 4 wherein monthly averages were derived from >50 sites which have valid data in all three modelled years. There is indication of nitrate load reductions in 2021, corroborating our modelling work herein as the concentrations in the early autumn-winter period (October to December) are much lower. The significantly higher sediment concentrations in 2016 between January and March (Fig 4) also support the increase in agricultural sediment loads predicted by CSM in 2016. The confirmed increasing sediment loads could be attributed to multiple interacting factors, including the sensitivity of sediment delivery to annual rainfall (higher later winter rainfalls are shown in Fig 4, expanding erosive crops and land uses, such as maize, as shown in Table 1 and, relatively lower uptake rates of sediment focussed mitigation measures as nutrient management related measures tend to be more regulatory in nature and thereby better enforced. More divergent trends were evident for phosphorus since continuous declines were observed at the monitored river sites (Fig 4) but no significant changes were computed using CSM. This is attributed to the dominance of the known significant reduction from non-agricultural sources, especially sewage treatment works in conjunction with phosphorus stripping. It is more challenging to evaluate the modelled emissions at national scale as not all farms were modelled and potential contributions from non-agricultural sources, e.g., channel banks, sewage treatment works and septic tanks were not quantified. Furthermore, the modelling work herein only estimated the potential emissions from three modelled years and ignored the residual effects associated with emissions in previous years which can persist in the form of a ‘memory effect’ [44,45]. These spatial and temporal mismatches inevitably hamper direct comparisons between monitored pollutant loads and modelled emissions at WMC scale.

While the research scope for this study focussed on the temporal changes of environmental impacts resulting from farm structural changes, the potential risks to agricultural production itself can be assessed with the estimated monetised values from the modelling outputs. The estimated relative change in agricultural production values from farming are provided in Supplementary information [40] and these suggest an increase of 14% and 10% relative to 2010 for 2016 and 2021, respectively. There is, however, inevitable spatial variability among the WMCs wherein up to 30% of the WMCs were predicted to experience more than a 5% reduction. This could mainly be attributed to the reduction in cattle numbers as shown in the Table 1.

The estimation of various indicators requires the quantification of selected pollutant loads and the specification of corresponding coefficients. The emission of pollutants from agricultural land involves interlinked physical, chemical and biological processes moderated by the interventions exerted by farming and mitigation activities. These lead to considerable spatial and temporal variability as shown in this study. These variabilities are further compounded by a multitude of off-farm events that are beyond farmers’ control. These include the increased frequency of extreme weather conditions, reductions in the area under permanent pasture, etc., which could increase overall national sediment loads (e.g., by >10% as shown in Table 3). Ongoing geopolitical instability and influential economic policy, e.g., tariffs, can lead to disruption to supply chains and volatile fertiliser markets. Increased fertiliser prices can lead to changes in farming practices, such as the reduced fertiliser application rates for most crops, especially on intensively managed farms (Table 2). At the same time, there is increased awareness of the climate change challenge among the general population and policy makers. This can drive tightened environmental regulations and the introduction of new environmental schemes (e.g., Environment Land Management) which can potentially reduce environmental footprints whilst the impacts can still vary spatially (c.f. [46]). These factors, and others, will all affect the future trends of environment footprints from the agricultural sector. Regardless, our work herein points to a general reduction in the environmental footprints of intensive farming across England between 2010 and 2021 in response to structural changes in the industry and reduced emission intensities of major farm inputs, e.g., fertiliser, electricity, etc. from upstream supply chains.

Quantification of the reduction in environmental footprints in tandem with structural changes in intensive farming across England since 2010 is timely in the context of current policy debates surrounding a Land Use Framework in England. The latter is envisaged by national policy teams as an instrument for ensuring improved delivery of food security in the context of meeting climate, nature and water quality targets. A recent public consultation in England considered a range of potential structural changes spanning small management changes (e.g., arable field margins riparian buffer strips) for business-as-usual farming (50,000 ha), incorporation of more trees alongside agricultural production, conversion of land use for climate and environmental benefits (e.g., short rotation coppice; 370,000 ha) and conversion away from agricultural production for delivering climate and environmental benefits (e.g., woodland creation, restoration of heathland habitats; 760,000 ha). Our modelling results, whilst not enacting the same management or conversion scenarios, point to the substantial reductions in environmental footprints achievable through structural change rather than improved management of business-as-usual farming systems. The need for such structural change planning has been underscored for some time given the more limited benefits of best management practices (e.g., [11]). Important challenges associated with further structural change in farms across England will be the need to protect yields from land used for high intensity farming alongside areas prioritised for biodiversity in the context of abiotic and biotic stresses and the need to improve yields from those areas of land which are currently under-performing for production. The latter could, on its own, deliver disproportionate benefits for national food security especially since the protection of production from the highest yielding areas will be challenging in the context of compound stresses and shocks.

Any modelling exercise has some associated limitations, including the specification of modelling domains, selection of temporal resolution, representation of key sources and processes due to data availability and accessibility at the right scale and coverage, limited understanding about the relevant processes and the development status of the model used. For the modelling domain our focus was on domestic land use and land management changes over the deep dive years, so the externalization of the GHG emissions associated with food imports from other countries still remains to be examined via a full LCA analysis. As mentioned before, the omission of specialised farms, with pigs or poultry will have had limited impacts on the computed national totals reported herein but they can be significant for localised emissions and unintended environmental impacts. The pollution of the River Wye from an intensive chicken farm [47] and the malpractices of pig farms in Norfolk [48] have recently highlighted these more localised issues. The availability of June Agriculture Survey data underpinning the CSM modelling framework inevitably constricted the temporal resolution; i.e., the number of modelled years which, in turn, precluded the application of any statistical methods for temporal trends in environmental footprints. Exploration of alternative data sources to make finer temporal representation and robust statistical analysis remains a research goal. Farm inputs and management are also constantly evolving and one significant development over the last decade has been the application of digestible wastes from manure-based or plant-based feedstocks onto agricultural land [49]. This specific and evolving nutrient application pathway is still not represented in the CSM modelling framework. Last, but not least, uncertainty analysis of the farm scale estimates and corresponding national aggregation procedures should also be undertaken. Such improvement outlined above would complement the findings reported herein.

## 5. Conclusions

Environmental footprints will keep changing in tandem with the transition of agriculture into a climate-resilient and economically-viable industry. With converging multiple global challenges, including climate change, energy supply and resource depletion, it is ever more important to explore potential mechanisms and pathways for effective, equitable and sustainable management of the unintended environment impacts from indispensable farming activities needed for a growing population. With increased demand for reliable evidence to support the continued reduction of environmental footprints from agricultural production throughout the world, regular strategic assessments using available modelling tools could play a significant role for informing both policy and management at national scale. A systematic approach combining multiple sectors and full supply chain consideration will help to produce even more reliable and trustworthy outcomes. Agricultural policy is typically delivered through a combination of regulation, incentivization and advice. Here, it is likely that all three instruments will be needed to help drive routine and strategic assessments of the environmental footprints of farm systems and importantly, by farmers and land managers themselves.
